# Therapeutic effect of hUC-MSCs from different transplantation routes on acute liver failure in rats

**DOI:** 10.3389/fmed.2025.1525719

**Published:** 2025-05-09

**Authors:** Yonghong Wang, Bei Wu, Yachao Tao, Menglan Wang, Dongbo Wu, Enqiang Chen, Hong Tang

**Affiliations:** ^1^Center of Infectious Diseases, West China Hospital of Sichuan University, Chengdu, China; ^2^Laboratory of Infectious and Liver Diseases, Institution of Infectious Diseases, West China Hospital of Sichuan University, Chengdu, China; ^3^Department of Hepatology, Public Health Clinical Center of Chengdu, Chengdu, China

**Keywords:** mesenchymal stem cells, cell transplantation, transplantation route, cell therapy, acute liver failure

## Abstract

**Objective:**

Acute liver failure (ALF) is a rare yet serious clinical syndrome. Recent studies have indicated that stem cells can effectively treat this condition. However, the optimal route for stem cell transplantation in the treatment of ALF remains unclear. This study aims to investigate the most effective transplantation route for stem cell therapy in ALF.

**Methods:**

Human umbilical cord mesenchymal stem cells (hUC-MSCs) expressing both luciferase and green fluorescent protein were generated using a lentiviral vector. The hUC-MSCs were transplanted via the tail vein, portal vein, and abdominal cavity. The survival and distribution of the transplanted hUC-MSCs in rats were assessed through *in vivo* imaging and immunofluorescence. Furthermore, the therapeutic effects of hUC-MSCs transplanted via different routes on ALF were compared.

**Results:**

The survival time of hUC-MSCs transplanted via the tail vein and portal vein was shorter compared to those transplanted intraperitoneally. The distribution of hUC-MSCs varied by transplantation route: those injected via the tail vein and portal vein were primarily found in the lungs and liver, respectively, while intraperitoneally transplanted hUC-MSCs predominantly localized in the abdominal cavity. In ALF rats, hUC-MSCs transplanted via the tail vein and portal vein improved survival rates, enhanced liver pathology, and reduced levels of inflammatory cytokines in liver tissue. In contrast, abdominal transplantation of hUC-MSCs showed no significant therapeutic effect.

**Conclusion:**

hUC-MSCs transplanted via the tail vein and portal vein exhibited similar therapeutic effects on ALF; however, abdominal transplantation of hUC-MSCs showed no significant effect.

## Introduction

ALF is a syndrome marked by the rapid decline of liver function, with clinical manifestations such as jaundice, coagulopathy, and hepatic encephalopathy, occurring after acute injury in patients without pre-existing chronic liver disease (1). Severe ALF carries a mortality rate of up to 40–50% (2). Currently, liver transplantation is the most effective treatment for ALF; however, due to a shortage of donor organs and the high costs associated with the procedure, only a limited number of patients are eligible for transplantation (3). Consequently, the search for new treatments for ALF remains a pressing clinical challenge. Stem cells have emerged as a promising new cell therapy, demonstrating potential in treating various diseases (4).

Stem cells possess self-replication capabilities, high proliferation rates, and the ability to differentiate into multiple cell types (5). Their therapeutic effects primarily stem from anti-inflammatory, anti-apoptotic, and immunomodulatory properties, as well as the secretion of growth factors and tissue repair mechanisms (6–8). Research on stem cells in liver diseases has predominantly focused on liver failure and liver fibrosis, with most studies indicating their beneficial effects on these conditions (9, 10). However, the application of stem cells for liver disease treatment lacks standardized criteria regarding cell source, dosage, transplantation route, and treatment duration (11). This absence of uniform guidelines may contribute to inconsistent efficacy of stem cell therapies in liver diseases. The optimal route for stem cell transplantation in ALF treatment remains uncertain. Therefore, this study aims to investigate the most effective transplantation route by comparing the therapeutic effects of hUC-MSCs administered via different routes in a rat model of ALF.

## Methods

### Culture and identification of hUC-MSCs

In our study, the third-passage hUC-MSCs were obtained from Hui Rong Tong Chuang Biological Technology Ltd. The complete culture medium used for hUC-MSCs was Dulbecco’s Modified Eagle’s Medium (DMEM) supplemented with 10% fetal bovine serum (Gibco, United States). The cells were incubated at 37°C in a 5% CO_2_ atmosphere, with the culture medium changed every 3–4 days.

hUC-MSCs from passages 3 ~ 9 were used in experiments. The characterization of hUC-MSCs was based on their morphology, cell surface marker expression, and differentiation potential. Flow cytometry (BD Accuri™ C6) was employed to assess the expression of CD90, CD105, CD34, and CD45 in hUC-MSCs, using PE-labeled antibodies purchased from BD. The differentiation potential of hUC-MSCs was evaluated using adipogenic and osteogenic differentiation kits (Cyagen Biosciences Inc., China), following the manufacturer’s protocols. Adipogenesis was assessed using Oil Red O staining, while osteogenesis was evaluated with Alizarin Red staining.

### hUC-MSCs luciferase labeling

Revive the cryopreserved third-passage hUC-MSCs and plate them when the cell confluency reaches 90%. Plate 1 × 10^5^ hUC-MSCs per well in a 6-well plate 24 h prior to viral infection. Dilute the Firefly Luciferase Lentifect™ (GeneCopoeia, United States) suspension to a multiplicity of infection (MOI) of 100 in complete medium containing polybrene at a final concentration of 6 μg/mL, and add 1.5 mL of this solution to each well. Incubate the plate in a 5% CO_2_ atmosphere at 37°C for 48 h, then transfer the cells to 10 cm culture dishes. Replace the old medium with fresh complete medium containing 1 μg/mL puromycin (Solarbio, China) daily until drug-resistant colonies emerge. Ultimately, the screened fifth-passage hUC-MSCs express luciferase and green fluorescent protein (GFP).

### hUC-MSCs transplantation and *in vivo* imaging

Luciferase-labeled hUC-MSCs were injected via the tail vein, portal vein, and peritoneal cavity, with an injection dose of 3 × 10^4^ cells/g and a concentration of 1 × 10^7^ cells/mL. At 0, 12, 24, and 48 h post-transplantation of hUC-MSCs, 150 mg/kg (15 mg/mL) of D-Luciferin potassium salt (Beyotime, China) was administered intraperitoneally. The fluorescence of the hUC-MSCs was then detected using the IVIS Spectrum 15 min after the D-Luciferin potassium salt injection.

### Animal models

Male SD rats (weighting 160–180 g) were obtained from Chengdu Dossy Experimental Animals Co., Ltd. (Chengdu, China). The rats were housed in an animal laboratory with stable temperature and humidity, with unrestricted access to chow and water. An ALF model was established through a single intraperitoneal injection of 800 mg/kg D-galactosamine (Sigma, United States) and 20 μg/kg lipopolysaccharide (Sigma, United States). hUC-MSCs transplantation was conducted 2 h after D-GalN/LPS treatment, with 3 × 10^4^ cells/g injected slowly over 3 to 5 min. Outcomes were assessed by comparing the 24-h survival rate, transaminase levels, and liver histopathology in the ALF rats.

### Biochemical analysis and HE staining

Blood samples were collected from each rat and centrifuged at 3,000 rpm for 15 min (Thermo, Germany) to obtain serum. The concentrations of alanine aminotransferase (ALT) and aspartate aminotransferase (AST) were measured using an automated biochemical analyzer (Abbott, United States). Each liver sample was fixed in 4% paraformaldehyde for 24 h prior to histological analysis. Fixed liver samples were then cut into small pieces, dehydrated, paraffin-embedded, and sectioned into 5 μm thick slices. These sections were stained with hematoxylin and eosin for pathological assessment.

### Real-time PCR analysis

Total RNA was extracted from liver tissue using the Trizol method (Invitrogen). Quantitative real-time PCR (qRT-PCR) was conducted using the LightCycler FastStart DNA Master PLUS SYBR Green I kit (Roche). The primer sequences (Table 1) specific to the target genes were synthesized by TSINGKE Biological Technology (Beijing, China). All samples were amplified in triplicate.

**Table 1:** Primer sequence of inflammation-related factors.

Primer name	5′-primer sequence-3′
IL10-F	CAGACCCACATGCTCCGAGA
IL10-R	CAAGGCTTGGCAACCCAAGTA
HGF-F	CATGAGAGAGGCGAGGAGAA
HGF-R	AAGTCACCTTGCCTTGATGG
TGFβ-F	ATACGCCTGAGTGGCTGTCT
TGFβ-R	TTCTCTGTGGAGCTGAAGCA
TNFa-F	GCTCCCTCTCATCAGTTCCA
TNFa-R	GCTTGGTGGTTTGCTACGAC
IL-1β-F	TACATCAGCACCTCTCAAGC
IL-1β-R	AGATTCTTCTGTCGACAATGC
IL6-F	CCAGTTGCCTTCTTGGGACT
IL6-R	AGTCTCCTCTCCGGACTTGT

### Statistical analysis

Data conforming to a normal distribution are presented as mean ± standard deviation, while data that do not conform to a normal distribution are expressed as median values. Categorical variables are reported as counts and percentages. Statistical analyses for normally distributed variables were performed using the *t*-test or analysis of variance (ANOVA), whereas the Mann–Whitney U test was applied for non-normally distributed variables and categorical variables. Statistical analyses were conducted using SPSS 17.0 software, and a *p*-value of < 0.05 (two-tailed) was considered statistically significant.

## Results

### Identification and labeling of hUC-MSCs

**Figure 1:**
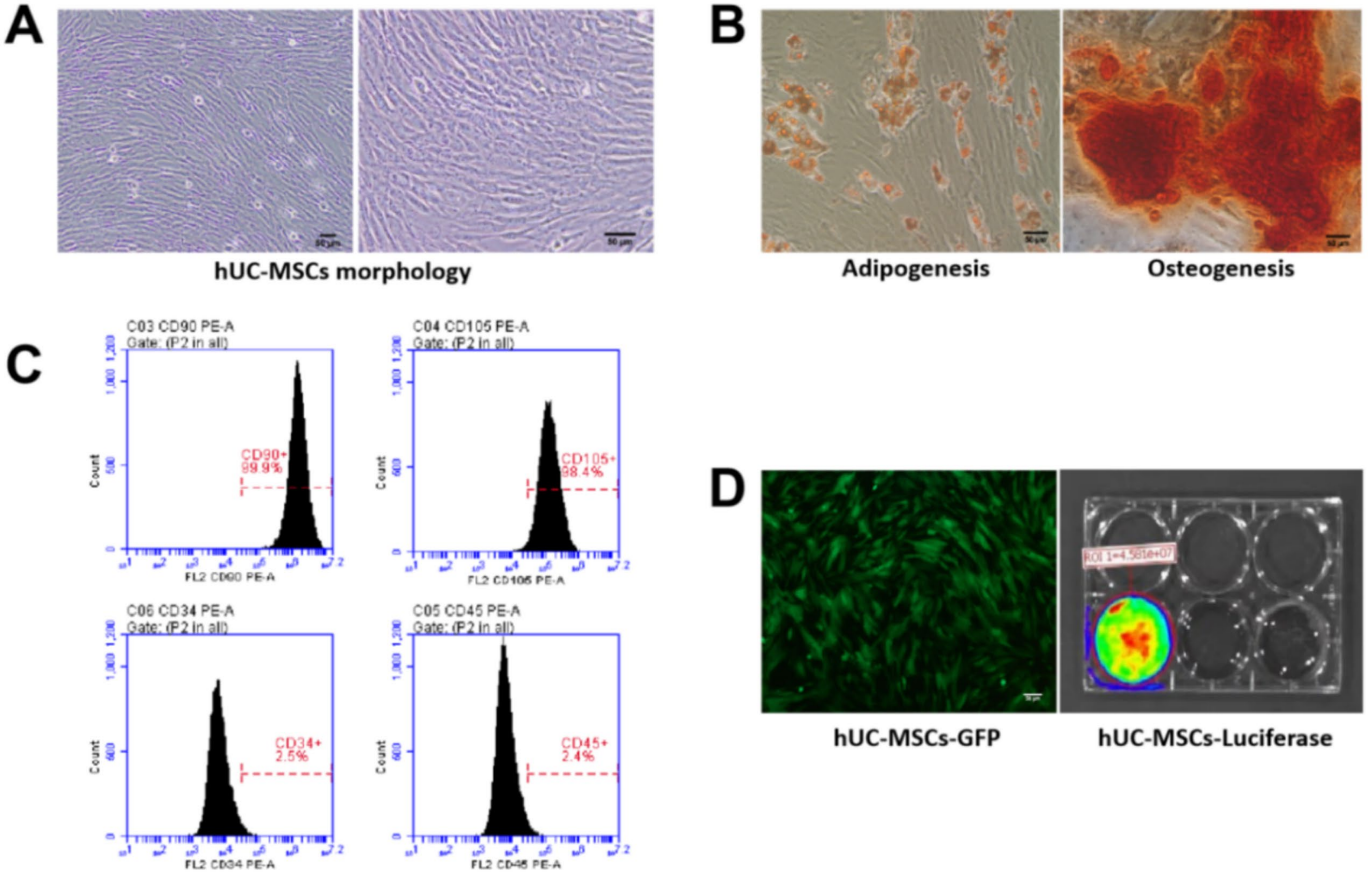
Identification and labeling of hUC-MSCs. **(A)** Observation of hUC-MSCs morphology under an optical microscope; **(B)** Detection of adipogenesis and osteogenesis capacity of hUC-MSCs; **(C)** Analysis of hUC-MSCs surface markers by flow cytometry; **(D)** The expression of GFP protein and luciferase in hUC-MSCs was detected.

Under normal culture conditions, hUC-MSCs adhered to plastic flasks, exhibiting clear cell morphology with either an epithelioid or spindle arrangement (Figure 1A). Following differentiation induced by a commercial stem cell osteoblast and adipocyte differentiation kit, hUC-MSCs were successfully induced to differentiate into osteoblasts and adipocytes (Figure 1B). The percentages of hUC-MSCs expressing the stem cell surface markers CD90 and CD105 were 99.9 and 98.4%, respectively, while the percentages of the negative surface markers CD34 and CD45 were 2.5 and 2.4%, respectively (Figure 1C). Additionally, hUC-MSCs selected using puromycin expressed both luciferase and GFP proteins (Figure 1D).

### Distribution and survival of hUC-MSCs transplanted via tail vein

**Figure 2:**
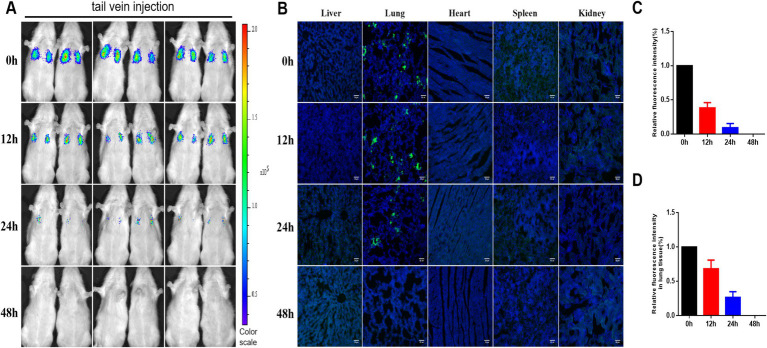
Distribution and survival of hUC-MSCs transplanted via tail vein. **(A)** The distribution and survival of hUC-MSCs transplanted via tail vein in rats were observed by *in vivo* imaging; **(B)** The distribution and survival of hUC-MSCs transplanted via tail vein in rats were observed by immunofluorescence; **(C)** Relative fluorescence intensity for *in vivo* imaging; **(D)** Relative fluorescence intensity of immunofluorescence.

Tail vein-injected hUC-MSCs rapidly accumulated in the lungs of rats, as observed through *in vivo* imaging. Following the injection of hUC-MSCs, the number of luciferase-labeled hUC-MSCs gradually decreased over time, with no detectable fluorescence signal at 48 h (Figures 2A,C). Immunofluorescence results corroborated the *in vivo* imaging findings, showing that GFP-labeled hUC-MSCs were primarily localized in the lungs, while no GFP-labeled hUC-MSCs were detected in the heart, liver, spleen, or kidneys (Figures 2B,D).

### Distribution and survival of hUC-MSCs transplanted via portal vein

**Figure 3:**
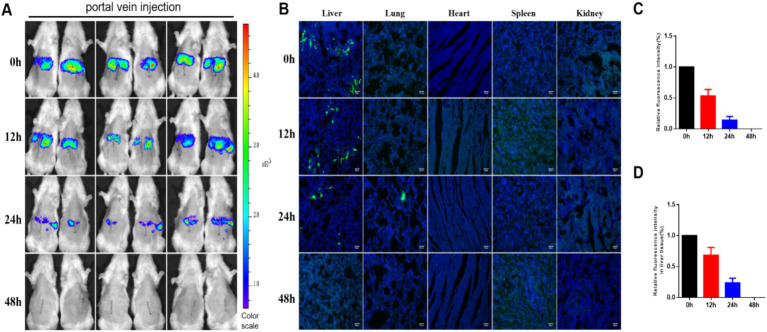
Distribution and survival of hUC-MSCs transplanted via portal vein. **(A)** The distribution and survival of hUC-MSCs transplanted via portal vein in rats were observed by *in vivo* imaging; **(B)** The distribution and survival of hUC-MSCs transplanted via portal vein in rats were observed by immunofluorescence; **(C)** Relative fluorescence intensity for *in vivo* imaging; **(D)** Relative fluorescence intensity of immunofluorescence.

Portal vein-injected hUC-MSCs rapidly accumulated in the liver of rats, as observed through *in vivo* imaging. Following the injection of hUC-MSCs, the number of luciferase-labeled hUC-MSCs gradually decreased over time, with no detectable fluorescence signal at 48 h (Figures 3A,C). Immunofluorescence results were consistent with the *in vivo* imaging findings, indicating that GFP-labeled hUC-MSCs were primarily localized in the liver, with a small number also present in the lungs at 24 h. No fluorescence signals were detected in the heart, spleen, or kidneys (Figures 3B,D).

### Distribution and survival of hUC-MSCs transplanted via abdominal cavity

**Figure 4:**
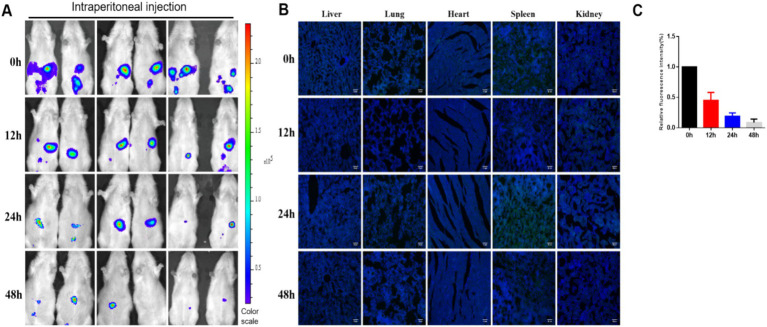
Distribution and survival of hUC-MSCs transplanted via abdominal cavity. **(A)** The distribution and survival of hUC-MSCs transplanted via abdominal cavity in rats were observed by *in vivo* imaging; **(B)** The distribution and survival of hUC-MSCs transplanted via abdominal cavity in rats were observed by immunofluorescence; **(C)** Relative fluorescence intensity for *in vivo* imaging.

Intraperitoneally injected hUC-MSCs accumulated in the abdominal cavity of rats, as observed through *in vivo* imaging. Following the injection, the number of luciferase-labeled hUC-MSCs gradually decreased over time; however, fluorescence signals were still detectable at 48 h (Figures 4A,C). Immunofluorescence detection revealed no distribution of GFP-labeled hUC-MSCs in the heart, liver, spleen, lungs, or kidneys at any of the four time points assessed (Figure 4B).

### Therapeutic evaluation of three routes of hUC-MSCs transplantation on ALF in rats

**Figure 5:**
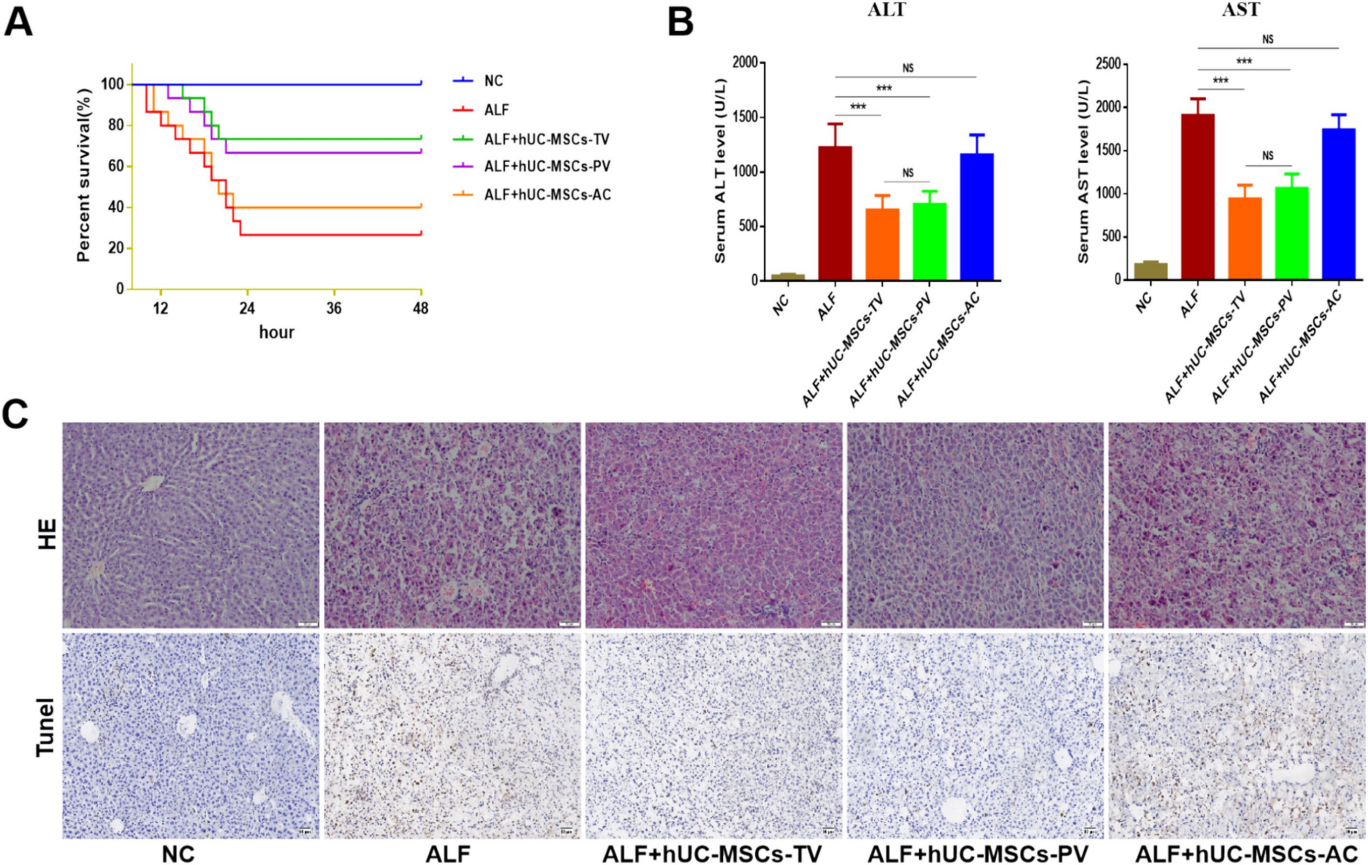
Therapeutic evaluation of three routes of hUC-MSCs transplantation on ALF in rats. **(A)** Survival curves of different hUC-MSCs treatment groups; **(B)** Serum ALT and AST levels of rats in different treatment groups; **(C)** HE and Tunel staining were used to observe the pathological changes of liver tissue. *p < 0.05; **p < 0.01; ***p < 0.001; NC: normal control; ALF: acute liver failure; hUC-MSCs-TV: hUC-MSCs transplanted via tail vein; hUC-MSCs-PV: hUC-MSCs transplanted via portal vein; hUC-MSCs-AC: hUC-MSCs transplanted via abdominal cavity.

**Figure 6:**
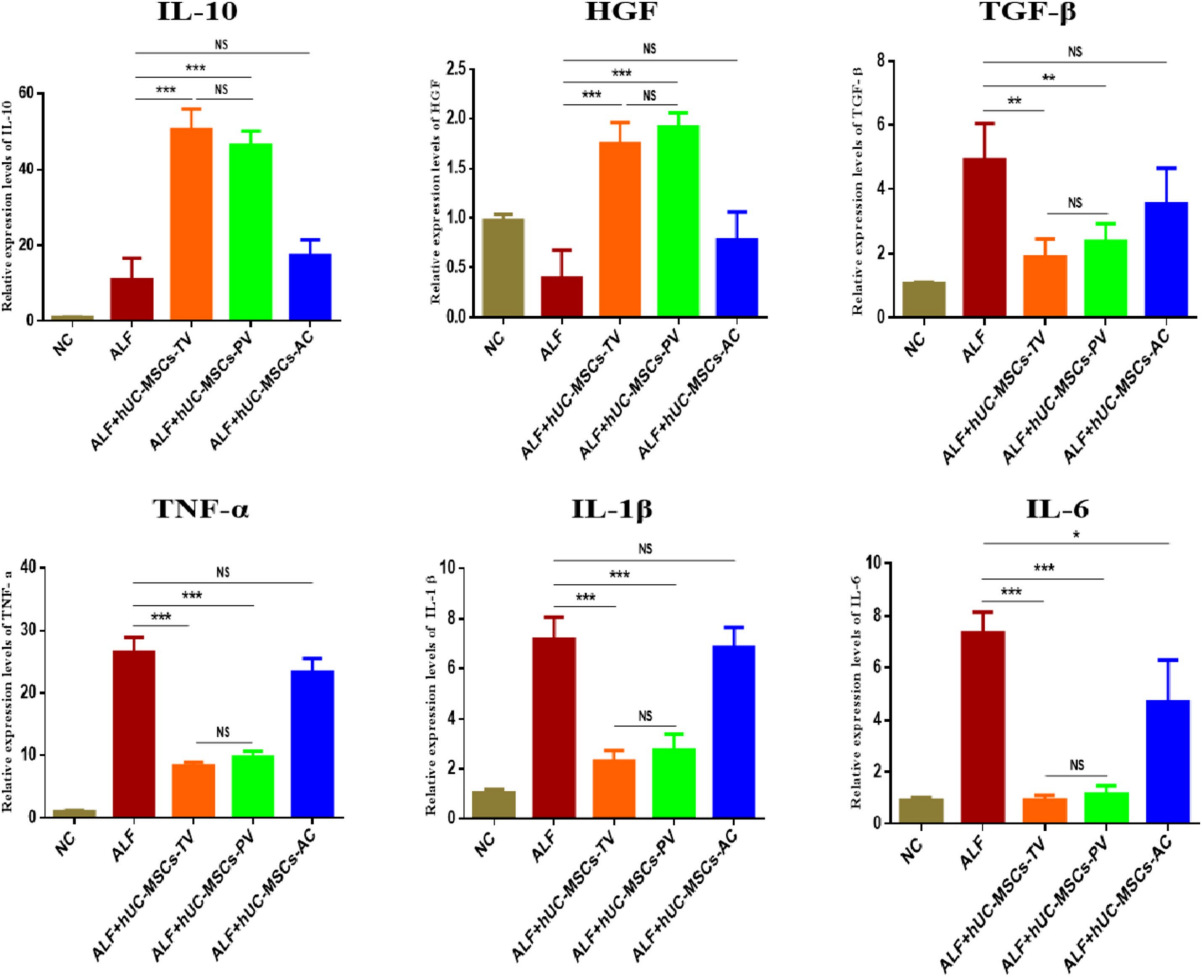
Detection of the expression of inflammation-related factors in liver tissue by Q-PCR. *p < 0.05; **p < 0.01; ***p < 0.001; NC: normal control; ALF: acute liver failure; hUC-MSCs-TV: hUC-MSCs transplanted via tail vein; hUC-MSCs-PV: hUC-MSCs transplanted via portal vein; hUC-MSCs-AC: hUC-MSCs transplanted via abdominal cavity.

In the rat ALF model established by D-GalN/LPS, survival rates (survival/total) after 48 h post-injection of PBS, hUC-MSCs-TV, hUC-MSCs-PV, and hUC-MSCs-AC were 4/15 (26.7%), 11/15 (73.3%), 10/15 (66.7%), and 6/15 (40%), respectively (Figure 5A). Liver function ALT/AST (IU/L) were 1,224 ± 108.9/1,909 ± 97.5, 653.2 ± 39.9/942.5 ± 47.9, 703.7 ± 38.5/1064 ± 52.8 and 1,157 ± 69.4/1,742 ± 66.3, respectively (Figure 5B). The main pathological changes observed in liver tissue included extensive hepatocyte necrosis, significant hepatic sinusoidal congestion, and widespread inflammatory cell infiltration in the ALF model. Both hUC-MSCs-TV and hUC-MSCs-PV improved the pathological changes of ALF, while hUC-MSCs-AC did not alleviate liver damage (Figure 5C). Additionally, quantitative PCR analysis of cytokine expression in liver tissue indicated that hUC-MSCs-TV and hUC-MSCs-PV significantly upregulated levels of IL-10 and HGF, while reducing levels of TGF-β, TNF-α, IL-1β, and IL-6 in ALF liver tissue. Although hUC-MSCs-AC reduced IL-6 expression, it had no significant effect on other cytokines (Figure 6). These results suggest that hUC-MSCs-TV and hUC-MSCs-PV exhibit similar therapeutic effects in treating ALF in rats, whereas hUC-MSCs-AC shows no significant benefit.

## Discussion

ALF is a rare yet severe clinical syndrome characterized by an extremely high mortality rate. The primary etiologies of ALF include viral hepatitis and drug-induced liver injury (12). Liver transplantation is the most effective treatment for acute liver failure; however, its widespread application is hindered by a shortage of donor livers and the high costs associated with the procedure (13). The mechanism underlying ALF can be divided into two stages. In the first stage, pathogens and toxins directly damage hepatocytes. In the second stage, the injured hepatocytes trigger excessive immune activation, resulting in immune-mediated liver damage (14). Therefore, eliminating pathogenic factors and inhibiting excessive immune activation are crucial components in the treatment of liver failure.

Stem cells have shown significant effects in a variety of liver diseases *in vivo* and *in vitro* (15, 16). Stem cells can be categorized into three main types: embryonic stem cells, adult stem cells, and induced pluripotent stem cells. Among these, mesenchymal stem cells (MSCs), a subtype of adult stem cells, are widely utilized in various applications (17). MSCs primarily contribute to disease treatment through tissue repair and immunomodulation, with immunomodulation being the most significant aspect (18). MSCs can inhibit the proliferation and activation of T cells, B cells, natural killer (NK) cells, and dendritic cells (DCs), thereby exerting an immunosuppressive effect (19). Consequently, MSCs may treat ALF by inhibiting over-activated immune responses. However, the optimal route for stem cell transplantation in the treatment of ALF remains unclear, making it crucial to select the most appropriate transplantation method.

In this study, the transplantation routes for stem cells included tail vein, portal vein, and intraperitoneal injection. Following the injection of hUC-MSCs via the tail vein and portal vein, the number of luciferase-labeled hUC-MSCs gradually decreased over time, with no detectable fluorescence signal observed after 48 h. In contrast, after intraperitoneal injection, while the luciferase-labeled hUC-MSCs also showed a gradual decline, fluorescence signals were still detectable at 48 h. These findings suggest that the survival time of hUC-MSCs transplanted via the tail and portal veins is shorter compared to those administered intraperitoneally. Additionally, most transplanted hUC-MSCs in rats were cleared within 24 h post-injection. This is consistent with previous studies indicating that MSCs injected through the tail vein in mice can be rapidly cleared within 24–48 h (20, 21). The *in vivo* clearance rate of MSCs needs to be considered when using MSCs to treat diseases.

The biodistribution of stem cells is largely influenced by the route of transplantation. Following tail vein transplantation, GFP-labeled hUC-MSCs were predominantly found in the lungs, with no detectable fluorescence signals in the liver, heart, spleen, or kidneys. In contrast, after portal vein transplantation, GFP-labeled hUC-MSCs were primarily distributed in the liver, with no fluorescence observed in the lungs, heart, spleen, or kidneys. Additionally, after intraperitoneal transplantation of GFP-labeled hUC-MSCs, no fluorescence signals were detected in the heart, liver, spleen, lungs, or kidneys. These findings suggest that stem cells injected peripherally via the intravenous route tend to be trapped in the lungs, with only a small fraction reaching other organs (22–24). However, portal vein transplantation allows the majority of stem cells to reside in the liver, which may be beneficial for stem cell treatment of liver diseases.

**Figure 7:**
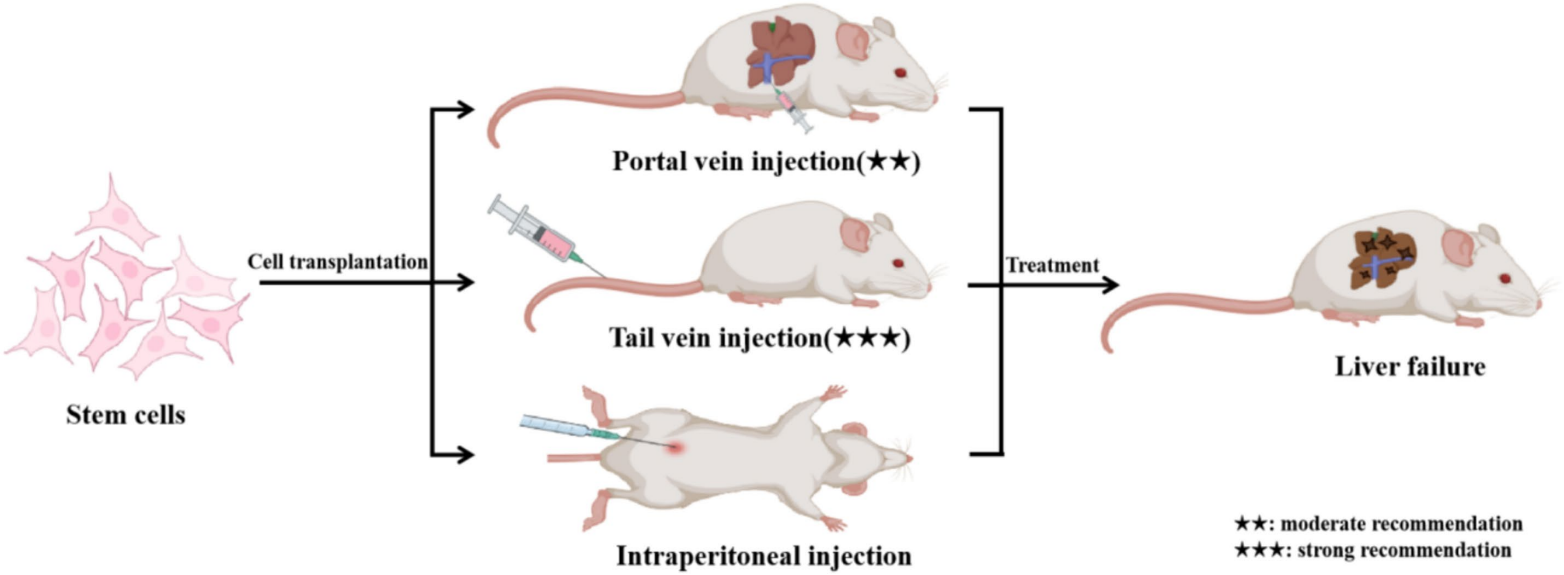
Stem cell transplantation via the portal vein, tail vein, and abdominal cavity for the treatment of liver failure. hUC-MSCs-TV and hUC-MSCs-PV have similar effects in the treatment of ALF in rats, but hUC-MSCs-AC has no obvious effect. Although hUC-MSCs transplanted via the tail vein and portal vein exhibit similar efficacy in treating ALF, portal vein transplantation is more invasive; therefore, the tail vein transplantation is more recommended.

It remains unclear whether the route of stem cell transplantation influences the outcomes of ALF. To address this, we compared the efficacy of hUC-MSCs transplanted via the tail vein, portal vein, and abdominal cavity in a D-GalN/LPS-induced ALF model in rats. The data indicate that both hUC-MSCs administered via the tail vein (hUC-MSCs-TV) and portal vein (hUC-MSCs-PV) exhibit similar therapeutic effects in treating ALF, while hUC-MSCs delivered via the abdominal cavity (hUC-MSCs-AC) showed no significant impact. Importantly, the biodistribution of hUC-MSCs in the lungs or liver did not correlate with the efficacy in ALF treatment, suggesting that the therapeutic effects of hUC-MSCs are systemic rather than localized. Similarly, Zheng et al. demonstrated that hUC-MSC transplantation via the tail vein has comparable therapeutic efficacy to intrahepatic injection (25). Sun et al. demonstrated that bone marrow-derived mesenchymal stem cells (BMSCs) have comparable effects on ALF when transplanted via the hepatic artery, caudal vein, or portal vein, while intraperitoneal transplantation showed no significant impact (26). Although hUC-MSCs transplanted via the tail vein and portal vein exhibit similar efficacy in treating ALF, portal vein transplantation is more invasive; therefore, the tail vein transplantation is more recommended (Figure 7).

Currently, the transplantation routes of MSCs to treat diseases are mainly divided into local and systematic ways (27). Local transplantation is the direct transfer of MSCs into the lesion, usually used for the treatment of myocardial infarction, osteoarthritis, and spinal cord injury (28). The systemic transplantation, which involves the transfer of MSCs through the bloodstream, is the most commonly used method. In this study, portal vein and tail vein transplantation of hUC-MSCs is a systematic way, while intraperitoneal injection is similar to local transplantation. MSCs exert their therapeutic effects primarily through directed differentiation to repair damaged tissues, secretion of reparative factors, and immune modulation (10, 29). However, many studies have shown that transplanted MSCs are difficult to repair damaged organs through differentiation (30). Therefore, the therapeutic effect of MSCs is mainly exerted by secreting cytokines, growth factors, chemokines and extracellular vesicles (31). In the treatment of ALF, both portal vein and tail vein transplantation are systemic approaches. hUC-MSCs delivered via the portal vein route can reach the liver more effectively, but there is no significant difference in therapeutic efficacy between the two transplantation pathways. The reason for this phenomenon may be that hUC-MSCs transplanted from portal vein and tail vein exert systemic therapeutic effects by secreting factors. The lack of therapeutic efficacy of intraperitoneally transplanted hUC-MSCs in ALF may be attributed to two factors: the intraperitoneal microenvironment potentially compromising the secretory function of hUC-MSCs, and the inability of hUC-MSCs-derived factors (e.g., exosomes) to penetrate the peritoneal barrier into the systemic circulation. Therefore, systemic transplantation is the best way to treat ALF with hUC-MSCs, especially through tail vein transplantation.

## Conclusion

hUC-MSCs-TV and hUC-MSCs-PV have similar effects in the treatment of ALF in rats, but hUC-MSCs-AC has no obvious effect. Although hUC-MSCs transplanted from the tail vein and portal vein have similar efficacy in liver failure, tail vein transplantation is more recommended.

## Data Availability

The original contributions presented in the study are included in the article/supplementary material, further inquiries can be directed to the corresponding authors.
